# DNA damage response in male gametes of *Cyrtanthus mackenii* during pollen tube growth

**DOI:** 10.1093/aobpla/plt004

**Published:** 2013-01-23

**Authors:** Tomonari Hirano, Keiichi Takagi, Yoichiro Hoshino, Tomoko Abe

**Affiliations:** 1 Innovation Center, RIKEN, 2-1 Hirosawa, Wako, Saitama 351-0198, Japan; 2 Graduate School of Advanced Science and Technology, Tokyo Denki University, 5 Senjyu-Asahicho, Adachi, Tokyo 120-8551, Japan; 3 Research and Development Department, Wakasa Wan Energy Research Center, 64-52-1 Nagatani, Tsuruga, Fukui 914-0192, Japan; 4 Field Science Center for Northern Biosphere, Hokkaido University, Kita 11, Nishi 10, Kita-ku, Sapporo 060-0811, Japan; 5 Nishina Center for Accelerator-Based Science, RIKEN, 2-1 Hirosawa, Wako, Saitama 351-0198, Japan

**Keywords:** DNA double-strand break, DNA repair, generative cell, heavy ion beam, pollen, spindle assembly checkpoint, sperm cell

## Abstract

Male gametophytes of plants are exposed to environmental stress and mutagenic agents during the double fertilization process and therefore need to repair the DNA damage in order to transmit the genomic information to the next generation. However, the DNA damage response in male gametes is still unclear. In the present study, we analysed the response to DNA damage in the generative cells of *Cyrtanthus mackenii* during pollen tube growth. A carbon ion beam, which can induce DNA double-strand breaks (DSBs), was used to irradiate the bicellular pollen, and then the irradiated pollen grains were cultured in a liquid culture medium. The male gametes were isolated from the cultured pollen tubes and used for immunofluorescence analysis. Although inhibitory effects on pollen tube growth were not observed after irradiation, sperm cell formation decreased significantly after high-dose irradiation. After high-dose irradiation, the cell cycle progression of generative cells was arrested at metaphase in pollen mitosis II, and phosphorylated H2AX (γH2AX) foci, an indicator of DSBs, were detected in the majority of the arrested cells. However, these foci were not detected in cells that were past metaphase. Cell cycle progression in irradiated generative cells is regulated by the spindle assembly checkpoint, and modification of the histones surrounding the DSBs was confirmed. These results indicate that during pollen tube growth generative cells can recognize and manage genomic lesions using DNA damage response pathways. In addition, the number of generative cells with γH2AX foci decreased with culture prolongation, suggesting that the DSBs in the generative cells are repaired.

## Abstract (precis)

During pollination and pollen tube growth, DNA damage is induced in the male gametes. However, detailed data on the DNA damage response is not available. Our results indicate that the generative cells of plants recognize and manage genomic lesions during pollen tube growth. In particular, we have shown that cell cycle progression of pollen mitosis II is strictly regulated by the spindle assembly checkpoint in response to genomic lesions.

## Introduction

In flowering plants, male gametophytes (pollen grains) transport pairs of sperm cells by elongating pollen tubes into female gametophytes (embryo sacs), which contain egg cells and central cells (reviewed in Lord and Russell 2002). The pollen can be categorized according to the number of cells in the pollen grain at the mature stage. Bicellular pollen contains a generative cell and a vegetative cell in the mature pollen, and sperm cells are derived by mitosis of the generative cell (pollen mitosis II; PMII), which takes place in the elongating pollen tube after germination. In tricellular pollen, a pair of sperm cells is formed in the mature pollen.

During pollination, pollen tube growth and double fertilization, the male gametophytes are exposed to environmental stress and mutagenic agents such as ultraviolet (UV) light and ionizing radiation (Jackson 1987). Therefore, DNA damage could be induced in the pollen grains or pollen tubes of the male gametes. Presumably, the male gametes are required to repair DNA damage for inheritance of the genetic information to occur and/or completion of PMII. When mature pollen from *Petunia hybrida* was UV-irradiated, unscheduled labelling of pollen DNA by ^3^H-thymidine was observed during pollen germination (Jackson and Linskens 1978). This observation suggests that a repair-like DNA synthesis is induced in the pollen DNA in response to UV irradiation. In *Lilium longiflorum*, the excision repair cross-complementation group 1 (*ERCC1*) gene has been isolated from a generative cell cDNA library (Xu *et al*. 1998), and ERCC1 is one of the proteins involved in the nucleotide excision repair pathway for removing DNA damage, including those induced by UV irradiation and chemical modification (de Laat *et al*. 1999). Transcriptome analysis in sperm cells has revealed that DNA repair or DNA damage response (DDR) genes are expressed in sperm cells, and the repair-related genes are enriched in the sperm cells compared with pollen (Engel *et al.* 2003; Borges *et al*. 2008). These findings indicate that DNA repair pathways function in male germline cells.

Heavy ion beams have been successfully applied as mutagens in plant breeding, as they can induce mutations at a high frequency and induce mutants with a broad spectrum of phenotypes even at low doses (Tanaka *et al*. 2010; Abe *et al*. 2012). Mature pollen grains from some species have been irradiated using heavy ion beams for genetic analysis, mutation breeding and the generation of new mutants (Nishimura *et al*. 1997; Kitamura *et al*. 2003; Naito *et al*. 2005; Fujita *et al*. 2012). The accelerated ions induce denser ionization in a more limited region than X-rays or γ-rays (Scholz 2006). Heavy ion irradiation can frequently induce DNA double-strand breaks (DSBs), which in turn develop deletion mutations (Shikazono *et al*. 2005; Kazama *et al*. 2011, Hirano *et al*. 2012). Mutations induced in mature pollen by ionizing radiation can be categorized as transmissible or non-transmissible mutations (Stadler and Roman 1948; Mottinger 1970; Vizir *et al*. 1994; Naito *et al*. 2005). In mutations induced by heavy ion irradiation of the pollen of *Arabidopsis thaliana*, extremely large deletions that are greater than several megabases are not often transmitted to the progeny (Naito *et al*. 2005).

As discussed above, DNA repair pathways in the male gametes are presumed to function during pollen tube growth. However, details of this process are not available. In the present study, we irradiated the bicellular pollen of *Cyrtanthus mackenii* with a heavy ion beam, which can induce DSBs. Since an *in vitro* culture system and techniques for male gamete isolation have been developed in the pollen of *C. mackenii* (Hirano and Hoshino 2010*a*), we analysed the DDR in the male gametes during pollen tube growth using the methods. We focused on cell cycle regulation of the generative cells and sperm cell formation in the pollen tubes. We have shown that after irradiation the cell cycle of the generative cells was arrested at metaphase by the spindle assembly checkpoint (SAC) in connection with recognition of DSBs and that the cell cycle progressed after completion of their repair. We reveal here that the pathways of DSB recognition and repair function in the male gametes of *C. mackenii* during pollen tube growth.

## Methods

### Plant materials and pollen culture

The *C. mackenii* plants used in the present study were grown in greenhouses. The anthers were collected from the flowers after dehiscence and maintained at −20 °C. In 1.5-mL tubes, anthers were irradiated with carbon ions (135 MeV per nucleon; corresponding to 22.5 keV µm^−1^ linear energy transfer in water) at a dose of 10–80 Gy and then stored at −20 °C. For pollen culture, the pollen grains from the irradiated or non-irradiated anthers were sown in 2 mL of liquid pollen culture medium (Hirano and Hoshino 2009) and cultured at 25 °C in the dark.

### Measurement of the pollen germination rate and sperm formation

We defined pollen germination as when the length of the pollen tube exceeds the size of the pollen grain (approximately>10 μm). We observed at least 500 pollen grains after 3 and 24 h of culture under an inverted microscope (IX-70; Olympus, Tokyo, Japan) and measured the germination rate. The lengths of 30 pollen tubes were measured after 24 h of culture. To visualize the nuclei within a pollen tube, 4′,6-diamidino-2-phenylindole (DAPI; final concentration, 1 μg mL^–1^) and 0.5 % Triton X-100 (final concentration) was added to the culture medium. After 15 min, the nuclei within pollen tubes were observed, and the proportion of pollen tubes containing two sperm nuclei was measured. At least 100 pollen tubes were measured and all experiments were repeated three times.

### Analysis of cell cycle phase and DNA damage

To observe the cell cycle phase in PMII and the distribution of phosphorylated histone H2AX (γH2AX) in the generative cells and sperm cells, immunofluorescence analysis was performed according to the methods described by Hirano and Hoshino (2010*a*) with several modifications. After 12 or 24 h of culture the generative cells or sperm cells in the pollen tubes were isolated and simultaneously fixed for 30 min in fixative containing 4 % (w/v) paraformaldehyde, 0.1 % (v/v) glutaraldehyde and 2 % (v/v) polyoxyethylene (20) sorbitan monolaurate in microtubule-stabilizing buffer. The cells were then transferred to a polylysine-coated coverslip using a microcapillary connected to a micropump (Nano Spuit; Ikeda Scientific Co. Ltd, Tokyo, Japan). The fixed cells were treated with a blocking agent (Image-iT FX Signal Enhancer; Molecular Probes, Inc., Eugene, OR, USA) for 60 min and then incubated with antibodies in phosphate-buffered saline supplemented with 0.05 % bovine serum albumin for 60 min at 37 °C. For γH2AX detection, we used 0.1 μg mL^–1^ anti-*A. thaliana* γH2AX rabbit polyclonal antibody (raised against C-terminal peptides of *Arabidopsis* H2AX; KGDIGSAS(p)QEF; Sigma Genosys Ltd, The Woodlands, TX, USA) and 5 μg mL^–1^ Alexa Fluor 488 goat anti-rabbit antibody (A11008; Molecular Probes). For microtubule staining, 1 μg mL^–1^ anti-α-tubulin mouse monoclonal antibody (A11126; Molecular Probes) and 5 μg mL^–1^ Alexa Fluor 546 goat anti-mouse antibody (A11003; Molecular Probes) were used. Thereafter, the cells were stained with 1 μg mL^–1^ DAPI for 15 min and mounted on coverslips in an antifade reagent (SlowFade Gold; Molecular Probes). Images were taken in 1.0-μm steps along the *Z*-axis by using a laser-scanning confocal microscope (FV-1000, Olympus). Each image stack was combined into a single image using Olympus FV10-ASW Ver. 3.1a. For this analysis, the number of generative cells and/or pairs of sperm cells used in each experimental plot was at least 90 from three independent experiments.

### Statistical analysis

The means of pollen tube length and the arcsin-transformed percentages of pollen germination, sperm cell formation and sperm abnormality were analysed using an *F*-test. The transformed values of the proportion of γH2AX foci-containing cells after 12 and 24 h of culture were compared using a *t*-test.

## Results

### Response of pollen grains to carbon ion irradiation

**Fig. 1:**
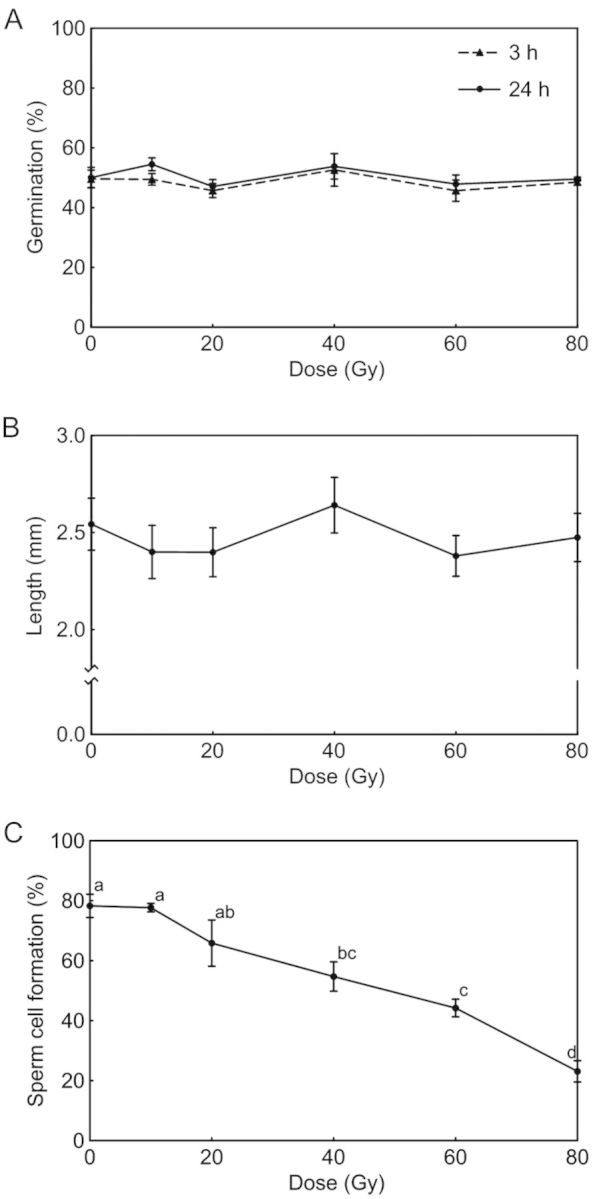
**Effects of carbon ion irradiation on pollen tube growth and sperm cell formation**. To analyse the effects of carbon ion irradiation, germination rates of irradiated pollen grains were counted at 3 and 24 h using an *in vitro* culture (A), and the length of the pollen tubes was measured at 24 h of culture (B). Sperm cell formation was defined as three nuclei observed in the pollen tube, and the proportion of pollen tubes containing three nuclei at 24 h of culture was calculated (C). Data are expressed as mean ± standard error for three individual experiments. Values with the same superscript letter are not significantly different (*P* < 0.05).

We first measured the germination rate of irradiated pollen grains. The germination rate of non-irradiated pollen grains reached a plateau at up to 3 h of culture, and the germination rate measured at 24 h was not higher than that measured at 3 h (Fig. 1A). The germination rates of pollen grains irradiated at 10–80 Gy were not significantly different (*P* < 0.05) from those of non-irradiated pollen grains at each time point. The pollen tube lengths of irradiated pollen grains after 24 h of culture also showed no decrease compared with those of non-irradiated pollen grains (Fig. 1B). Therefore, the range of irradiation doses used in this study had no inhibitory effects on pollen tube growth. Because *C. mackenii* forms bicellular pollen, we focused on sperm cell formation during pollen tube growth. In non-irradiated pollen grains, the rate of sperm cell formation in 24-h cultured pollen tubes was 78 % (Fig. 1C). Sperm formation rates at >40 Gy irradiation decreased significantly compared with those of non-irradiated pollen grains; when pollen grains were irradiated at 40 and 80 Gy, sperm formation rates were 55 and 23 %, respectively (Fig. 1C).

### Cell cycle arrest in PMII

**Fig. 2:**
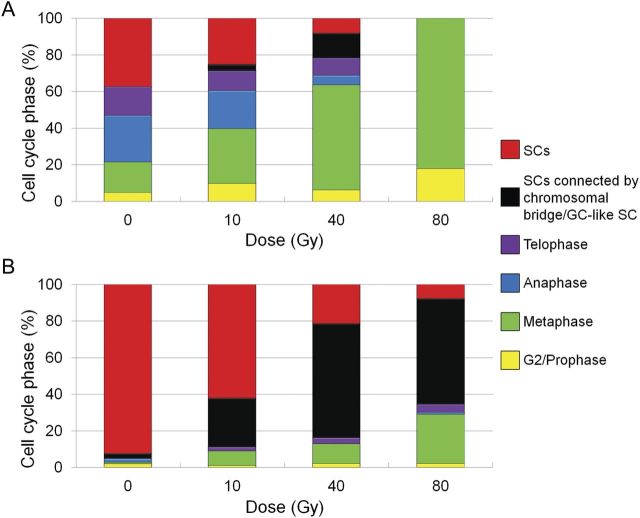
**Change of cell cycle phase in PMII after carbon ion irradiation**. The phase of the male gametes isolated from the pollen tubes cultured for 12 h (A) and 24 h (B) based on the microtubule array in the cells. The categories of sperm cells (SCs) consist of normal SCs and SCs with lagging chromosomes. Male gametes that completed PMII, with a pair of SCs, SCs connected by a chromosomal bridge or a generative-cell-like sperm cell (GC-like SC) were counted as one male gamete, and proportions of the cell cycle phase were based on the number of male gametes from three independent experiments.

Sperm formation in *C. mackenii* begins at 9–12 h of culture (Hirano and Hoshino 2010*a*). For a detailed analysis of sperm cell formation, we investigated the cell cycle phase in PMII of irradiated pollen grains after 12 h of culture by isolating male gametes from the pollen tubes. The cell cycle phases of generative cells in mature pollen are not strictly defined (Palevitz and Tiezzi 1992; Palevitz 1993). In mature pollen of *C. mackenii*, the generative cells have 2C of DNA content (Hirano and Hoshino 2010*a*), indicating that the generative cells have already finished DNA replication toward PMII at the stage. Therefore, we defined the phase of the generative cell before metaphase as G2/prophase in this study. In non-irradiated pollen grains, 78 % of generative cells completed metaphase (Fig. 2A). However, the majority of the generative cells irradiated at 40 and 80 Gy arrested at metaphase, and the number of generative cells that completed metaphase was 36 and 0 % after irradiation with 40 and 80 Gy, respectively (Fig. 2A). To analyse the behaviour of the arrested cells, we also investigated the cell cycle phase after 24 h of culture. In this culture period, most of the generative cells in non-irradiated pollen grains had formed pairs of sperm cells (Fig. 2B). The cell cycle in irradiated generative cells after 24 h of culture progressed in comparison with that after 12 h of culture, and the proportions of the generative cells that had completed metaphase after 24 h of culture were 87 % at 40 Gy and 71 % at 80 Gy (Fig. 2B).

### Sperm abnormality

**Fig. 3:**
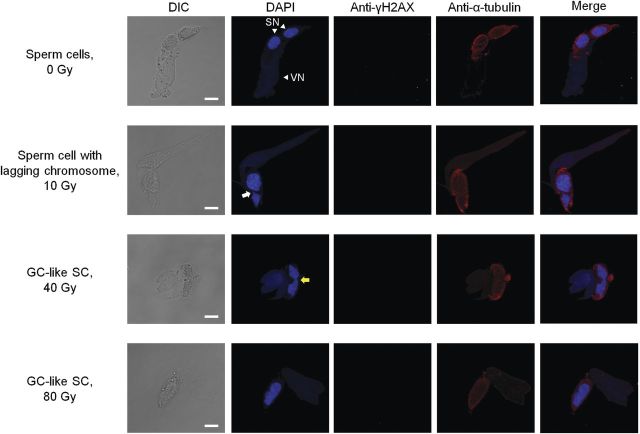
**Abnormalities in the sperm nuclei derived from irradiated pollen grains**. The male gametes were isolated from the pollen tubes cultured for 24 h. The merged images show nuclei stained with DAPI (pseudocolour: blue), γH2AX stained with anti-γH2AX antibody followed by secondary antibody (pseudocolour: green) and microtubules stained with anti-α-tubulin antibody followed by secondary antibody (pseudocolour: red). The white and yellow arrows indicate lagging chromosome and chromosomal bridges, respectively. VN, vegetative nucleus; SN, sperm nucleus. Bars = 10 μm.

**Fig. 4:**
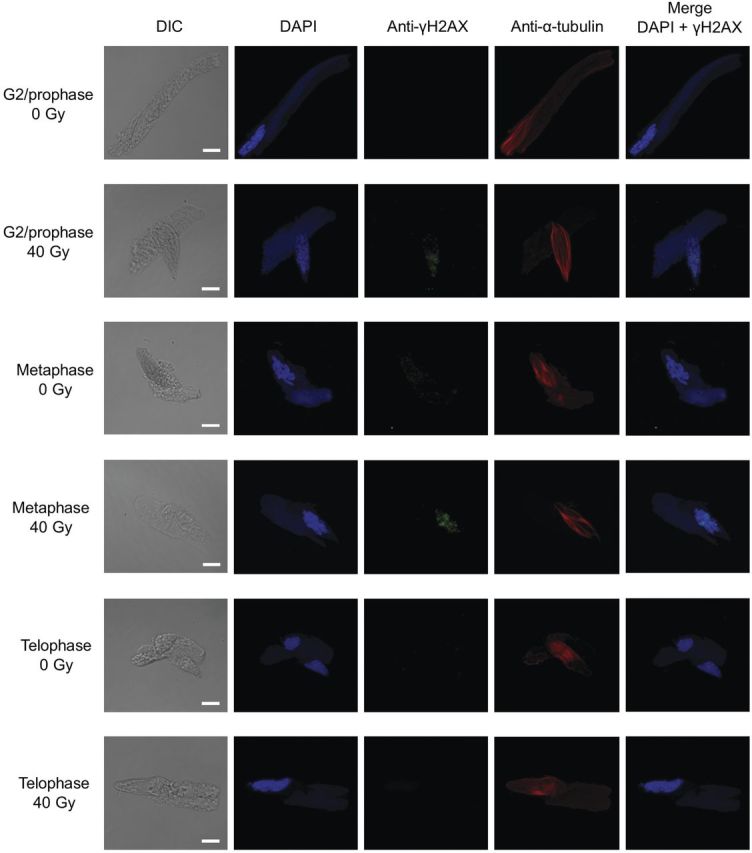
**Detection of γH2AX in the nuclei of male gametes**. The male gametes were isolated from the pollen tubes cultured for 12 h. Merged images show nuclei stained with DAPI (pseudocolour: blue) and γH2AX stained with anti-γH2AX antibody followed by secondary antibody (pseudocolour: green). Bars = 10 μm.

After carbon ion irradiation, sperm abnormalities such as lagging chromosomes and asymmetric division were observed (Fig. 3). After 24 h of culture, the proportion of sperm cell pairs with lagging chromosomes in the PMII-completed male gametes was particularly high after irradiation with 10 Gy (Table 1). The male gametes from irradiated pollen grains contained generative-cell-like nuclei and sperm-cell-like microtubule arrays (Fig. 3). The cells, which contained chromosomal bridges and did not show a clear chromosome separation, could progress into telophase (Fig. 4). Therefore, this suggests that the cells with generative-cell-like nuclei and sperm-cell-like microtubule arrays completed PMII but failed in chromosome separation. We defined the cells as generative-cell-like sperm cells (GC-like SCs). The sperm cells connected by a chromosomal bridge and the GC-like SCs were observed beginning at 12 h of culture (Fig. 2B), and male gametes in that category amounted to 24, 74 and 90 % of the PMII completed male gametes irradiated with 10, 40 and 80 Gy, respectively, at 24 h of culture (Table 1).

**Table 1:** **Effect of irradiation doses on abnormal sperm formation after 24 h of culture**. The male gametes used for this analysis were isolated from the pollen tubes cultured for 24 h. Each value is expressed as the mean ± standard error for three individual experiments. In each column, values with the same superscript letter are not significantly different (*P* < 0.05).

Dose (Gy)	Pair of SCs (%)		SCs connected by chromosomal bridge and GC-like SC (%)
	Normal	With lagging chromosome	
0	96.0 ± 2.1^a^	1.2 ± 1.2^a^	2.8 ± 1.5^a^
10	53.4 ± 13.1^b^	22.2 ± 3.5^b^	24.4 ± 10.9^b^
40	21.0 ± 7.5^c^	5.3 ± 5.3^a^	73.6 ± 12.8^c^
80	8.6 ± 1.5^c^	2.0 ± 2.0^a^	89.5 ± 0.5^c^

SC, sperm cell; GC-like SC, generative-cell-like sperm cell.

### DNA repair in male gametes during pollen tube growth

We hypothesized that the DNA damage in the generative cells irradiated with high doses was not completely repaired even after 12 h of culture. In the male gametes derived from the pollen grains irradiated at 10–80 Gy, γH2AX foci (as an indicator of DSBs) were not detected in anaphase, telophase or sperm cells, irrespective of whether they contained chromosomal aberrations (Fig. 4, Table 2). However, the foci were observed in G2/prophase and metaphase cells (Fig. 4), and the proportions of male gametes with these foci were 41 % at 40 Gy and 83 % at 80 Gy after 12 h of culture (Table 2). Therefore, we concluded that the DSBs induced by carbon ion irradiation remained in most of the cells arrested during PMII progression. After doses of 40 and 80 Gy, the proportion of male gametes with γH2AX foci decreased with prolonged culture period (24 h).

**Table 2:** **Proportion of male gametes with γH2AX foci**. Male gametes that completed PMII (pairs of sperm cells, sperm cells connected by a chromosomal bridge or GC-like SCs) were counted as one male gamete, and the numbers of male gametes in each phase were summed from three independent experiments. The percentage of male gametes with γH2AX foci is expressed as mean ± standard error.

Dose (Gy)	Culture period (h)	Percentage of male gametes with γH2AX foci with respect to total male gametes	The number of male gametes in each phase			
			G2/prophase and metaphase		From anaphase to PMII-completed cells	
			With γH2AX foci	Total	With γH2AX foci	Total
0	12	0.0 ± 0.0	0	22	0	80
	24	0.8 ± 0.8^NS^	1	3	0	101
10	12	1.3 ± 1.3	1	36	0	55
	24	7.5 ± 5.3^NS^	5	6	0	84
40	12	41.2 ± 4.0	40	61	0	35
	24	6.6 ± 0.7*	6	12	0	93
80	12	83.0 ± 3.5	82	100	0	0
	24	23.1 ± 8.0*	22	26	0	64

NS, not significant.

*The percentages of each dose at 12 and 24 h of culture were significantly different (*P* < 0.05).

## Discussion

### DNA damage response in the male gametes during pollen tube growth

In the present study, we attempted to analyse the DDR in male gametes during pollen tube growth. When DSBs were induced in the generative cells by carbon ion irradiation, DNA damage was detected as γH2AX foci in the cells (Fig. 4). H2AX is a variant form of the histone H2A, and H2AXs that surround a DSB are phosphorylated mainly by protein kinase ATM (ataxia telangiectasia mutated; Löbrich *et al*. 2010). Ataxia telangiectasia mutated is activated by DSBs (Lee and Paull 2005) and mediates signal transduction pathways for the DDR (Garcia *et al*. 2003; Culligan *et al*. 2006). The detection of γH2AX foci in the irradiated generative cells implies that the pathway still operates in the generative cells during the transport process to female gametophytes. Using the methods described previously (Hirano and Hoshino 2010*a*), we isolated cells as male germ units in which the generative cell or the sperm cell pair was closely associated with the vegetative nucleus. Although γH2AX was also investigated in the vegetative nuclei after 12 and 24 h of culture, no foci were detected (Figs 3 and 4). This suggests the possibility that DSB recognition and/or DSB repair strategy are functioning in the generative cells but not in the vegetative cells. In the pollen of *L. longiflorum*, male-gamete-specific histone genes, including *H2A*, have been discovered, and the histone genes are expressed in the generative cell but not in the vegetative cell and the somatic cell (Xu *et al.* 1999; Ueda *et al.* 2000). It is inferred that one of the reasons for vegetative nuclei without the foci is the difference in histone usage between the generative nucleus and the vegetative nucleus, and ATM cannot phosphorylate the histone distributed in the vegetative nucleus. In the pollen of *A. thaliana*, the DSB repair genes, such as RAS associated with diabetes protein 51 (*Rad51*) and Ku70 homologue, are enriched in the sperm cells compared with pollen (Borges *et al*. 2008). Moreover, the mutagenic activity of transposable elements is suppressed in sperm cells by siRNA from transposable-element-activated vegetative nuclei (Slotkin *et al*. 2009). Taken together, it is indicated that the maintenance mechanisms for genomic stability are not functioning equally in the generative cells and vegetative cells. Further experimental evidence is needed to clarify this hypothesis on the DDR for DSBs in the male gametophyte.

### Control of cell cycle progression in PMII

The G2 to prophase of mitotic transition (G2/M) has been identified as a DNA damage checkpoint that occurs during G2 phase (Waterworth *et al.* 2011). Cell cycle machinery is controlled by complexes of cyclin and cyclin-dependent kinases (CDKs), and one of the CDKs, CDKA;1, acts at the G1/S transition and in G2 (Francis 2007). *WEE1* expression is strongly induced by DSBs in an ATM-dependent manner, and WEE1 leads to inhibition of CDKA;1 kinase activity by tyrosine phosphorylation and arrests cells in the G2 phase (De Schutter *et al*. 2007). In this study, there was little evidence for G2/M arrest and the majority of irradiated generative cells proceeded into metaphase of mitosis (Fig. 2). Because γH2AX foci were detected in the irradiated generative cells, an ATM-dependent signalling cascade is predicted to function after DSB recognition. Taken together, it can be interpreted that the cell cycle phase of the generative cells in mature pollen is already beyond the G2/M checkpoint, such as prophase.

As a checkpoint for fidelity of chromosomal segregation in the mitotic phase, the SAC is activated by abnormalities of chromosomal tension due to kinetochore–spindle attachments and/or occupancy of the kinetochore by microtubules, and dividing cells with abnormalities are arrested at metaphase until all chromosomes achieve bipolar attachment (Musacchio and Salmon 2007). Unattached kinetochores contribute to arrest of anaphase progression by inhibition of anaphase-promoting complex/cyclosome activity. In plants, SAC components have been characterized at the unattached kinetochores (Yu *et al*. 1999; Kimbara *et al*. 2004; Caillaud *et al*. 2009). The generative cells irradiated with high doses (40 and 80 Gy) were arrested at metaphase, and the cell cycle arrest is released after a prolonged culture period (Fig. 2). When γH2AX foci in irradiated generative cells were analysed, foci were detected in the majority of the arrested cells (Table 2). However, the cells passing through metaphase contained no γH2AX foci. In *A. thaliana*, it has been reported that DSBs in anaphase nuclei can be detected by anti-γH2AX antibody (Friesner *et al.* 2005). Therefore, it is suggested that the generative cells with chromosomal lesions, such as DSBs, were arrested in the cell cycle progression by the SAC until completion of its repair. Moreover, aberrant generative cells in telophase with chromosomal bridges and unclear chromosomal separation were detected (Fig. 4), thereby indicating that after DSB repair generative cells progress into anaphase and telophase, and do not skip the cell cycle phase in PMII.

### DNA repair in the male gametes

Double-strand breaks are repaired by at least four pathways: homologous recombination (HR), canonical non-homologous end-joining (NHEJ), alternative NHEJ and single-strand annealing (SSA; Ciccia and Elledge 2010). Male gametes with γH2AX foci decreased with prolonged culture time (Table 2). Therefore, DSBs in the generative cells were considered to be repaired by one of the above pathways. Heavy ion irradiation can induce chromosomal rearrangements in addition to deletion mutations (Shikazono *et al*. 2005; Kazama *et al.* 2011; Hirano *et al*. 2012). It has been reported that Rad51, which is required for strand invasion into a homologous sequence during HR, was prevented from binding to the resected end of DSBs in chromosomes of vertebrates in the M phase (Ayoub *et al*. 2009; Peterson *et al*. 2011). In actuality, both chromosomal and cell division abnormalities were observed in an irradiation-dose-dependent manner in the PMII-completed cells (Table 1), suggesting that non-allelic HR (Liu *et al*. 2012) or error-prone pathways such as canonical NHEJ, alternative NHEJ or SSA were used for DSB repair in the generative cells. Signalling cascades after DSB induction and the repair pathways of DSB in mitosis are still unclear in plants. The DDR during mitosis should be investigated further, including whether the DDR in generative cells differs from that in somatic cells.

## Conclusions and forward look

As H2AXs surrounding the DSBs were phosphorylated in the generative cells, the DNA-damage-sensing process was active during sperm cell formation. Moreover, the generative cells containing DNA lesions induced by the carbon ion beam were regulated in the cell cycle progression of PMII by SAC at metaphase rather than at the G2/M boundary. Therefore, the generative cells recognized and managed genomic lesions during the transport process to female gametophytes. This is a unique experimental system that combines *in vitro* cultures of bicellular pollen and micromanipulation techniques (Hirano and Hoshino 2010*b*), and analysis of the generative cells after induction of DNA damage for the investigation of DDR during mitosis in plants is possible. Utilization of this system may lead to a better understanding of the DDR during mitosis.

## Sources of funding

This research was supported by a grant received from the Japan Society for the Promotion of Science (JSPS) through the ‘Funding Program for Next Generation World-Leading Researchers (NEXT Program)’, initiated by the Council for Science and Technology Policy (CSTP), JSPS KAKENHI Grant Number 22780034 and the Social Infrastructure Technology Department Program of RIKEN.

## Contributions by the authors

T.A. coordinated the project and obtained the beam times. T.H. and T.A. participated in the design of the study. T.H. and T.A. performed the carbon ion irradiation. T.H., K.T. and Y.H. carried out the cytological analyses. T.H. and T.A. were primarily responsible for drafting and revising the manuscript with contributions from the co-authors. All authors read and approved the final manuscript.

## Conflict of interest statement

None declared.
